# Social and demographic factors associated with receipt of a COVID-19 vaccine initial booster dose and with interval between primary series completion and initial booster dose uptake among persons aged ≥ 12 years, United States, August 2021-October 2022

**DOI:** 10.1016/j.vaccine.2024.02.089

**Published:** 2024-03-07

**Authors:** Lu Meng, LaTreace Harris, Lauren Shaw, Hoody Lymon, Hannah Reses, Jeneita Bell, Peng-Jun Lu, Lynn Gibbs-Scharf, Terence Chorba

**Affiliations:** a CDC COVID-19 Response Team, USA; b Division of Health Quality Promotion, National Center for Emerging and Zoonotic Infectious Diseases, 1600 Clifton Rd NE, Centers for Disease Control and Prevention, Atlanta, GA, 30329, USA; c Immunization Services Division, National Center for Immunization and Respiratory Diseases, 1600 Clifton Rd NE, Centers for Disease Control and Prevention, Atlanta, GA, 30329, USA; d Division of Tuberculosis Elimination, National Center for HIV, Viral Hepatitis, STD, and TB Prevention, 1600 Clifton Rd NE, Centers for Disease Control and Prevention, Atlanta, GA, 30329, USA

**Keywords:** COVID-19, Coronavirus, Booster dose, COVID-19 vaccination

## Abstract

COVID-19 booster dose vaccination has been crucial in ensuring protection against COVID-19 including recently predominant Omicron variants. Because vaccines against newer SARS-CoV- 2 variants are likely to be recommended in future, it will be valuable to understand past booster dose uptake among different demographic groups. Using U.S. vaccination data, this study examined intervals between primary series completion and receipt of first booster dose (monovalent or bivalent) during August 2021 - October 2022 among persons ≥12 years of age who had completed a COVID-19 vaccine primary series by October 2021. Sub-populations who were late booster recipients (received a booster dose ≥12 months after the primary series) or received no booster dose included persons <35 years old, Johnson & Johnson/Janssen vaccine primary dose recipients, persons in certain racial and ethnic groups, and persons living in rural and more socially vulnerable areas, and in the South region of the United States; these groups may benefit the most from public health outreach efforts to achieve timely COVID-19 vaccination completion in future.

## 1. Introduction

COVID-19 booster dose vaccination has been crucial in ensuring maximal protection against COVID-19 including recently predominant Omicron variants. Prior to bivalent booster dose recommendations released in September 2022, it was recommended that eligible persons aged ≥ 5 years receive a monovalent booster dose ≥ 2 months after vaccination with the 1-dose Johnson & Johnson’s Janssen vaccine primary series or ≥ 5 months after the second dose of the Pfizer-BioNTech or Moderna mRNA vaccines 2-dose primary series [1,2]. Monovalent booster dose consisted of an mRNA component corresponding to the original SARS-CoV-2 strain; bivalent mRNA booster dose included the original component and an mRNA component corresponding to Omicron variant BA.4 and BA.5 lineages authorized by the U.S. Food and Drug Administration (FDA) on August 31, 2022 [2,3]. On September 24, 2021, the U.S. Centers for Disease Control and Prevention (CDC) first recommended a monovalent booster dose of the Pfizer-BioNTech COVID-19 vaccine for certain populations and for those in high risk occupational and institutional settings [4,5]. Booster dose recommendations were expanded to all eligible adults (aged ≥ 18 years) in November 2021, and later expanded to include additional age groups, including adolescents aged 12–17 years starting in January 2022 [5]. On September 1, 2022, CDC updated COVID-19 vaccination recommendations for the use of bivalent booster from Pfizer-BioNTech for people aged ≥ 12 years and from Moderna for people aged ≥ 18 years [6]. However, many people did not receive a booster dose or delayed receiving a booster dose [7,8].

Primary series COVID-19 vaccine uptake has been high for most adult populations; however, nearly 80 million persons aged ≥ 12 years who were eligible to receive a booster dose had not received one by October 31, 2022 [9]. Because COVID-19 vaccines against newer SARS-CoV-2 variants are likely to be recommended in future, as happened on September 12, 2023, it will be valuable to understand past booster dose uptake, including intervals between previous doses and among different demographic groups [10,11]. The purpose of this study was to understand the timing of COVID-19 booster dose (monovalent or bivalent) administration during August 1, 2021-October 31, 2022 among U.S. eligible persons aged ≥ 12 years who completed the primary series prior to October 31, 2021. In addition, this study examined demographic factors associated either with non-receipt of an initial booster (monovalent or bivalent) dose or with receipt of a booster dose ≥ 12 months after primary series completion.

## 2. Methods

Over 263 million de-identified COVID-19 vaccine records among persons ≥ 12 years of age in the U.S. (records of 168 million primary series vaccinations administered from December 14, 2020 through October 31, 2021 and 95 million initial booster dose administered from August 1, 2021 through October 31, 2022) were reported to CDC from 49 states and the District of Columbia (DC) by state and local health departments, pharmacies, and federal entities, principally the Department of Veterans Affairs, the Department of Defense, and the Indian Health Service; these data were reported through immunization information systems (IISs), the Vaccine Administration Management System (VAMS), or through direct data submission.^a^ These data included persons who received a booster dose in clinical trials prior to September 24, 2021, thus August 1, 2021 was selected as the initial date limit for booster dose administration in this study. Vaccination records were analyzed using the cloud-based data platform Microsoft Azure Data-Bricks (Azure Databricks ∣ Microsoft Azure). De-identified primary series vaccination data and initial booster dose data were matched according to a unique recipient number assigned by the reporting entity and an 8–12–digit reporting source code. Initial boosters administered during August 1, 2021-October 31, 2022 included both monovalent and bivalent boosters.

Two logistic regression models were developed to examine (1) factors associated with non-receipt of a booster (monovalent or bivalent) dose among persons who had completed a COVID-19 vaccine primary series by October 31, 2021 and (2) factors associated with receipt of an initial booster dose (monovalent or bivalent) ≥ 12 months after primary series completion among those who had completed the primary series by October 31, 2021. Characteristics assessed included primary series vaccine product brand (Moderna, Pfizer-BioNTech, Janssen), age group (12–17, 18–34, 35–49, 50–64, ≥65 years), sex (male, female), race and ethnicity (Hispanic/Latino, Non-Hispanic Black, Non-Hispanic American Indian/Alaska Native, Non-Hispanic Asian/Other Pacific Islander, Non-Hispanic White, Other/multiracial/unknown), region (South, Midwest, Mountain, Pacific, Northeast),^b^ urbanicity^c^ (large central metro, large fringe metro, medium metro, small metro, micropolitan, non-core) [12], and CDC/ATSDR Social Vulnerability Index (SVI) of county of residence (low, medium, and high) [13]. Factors included in SVI scores include socioeconomic status, household composition, disability, minority status, housing types, and transportation in the county. A lower SVI score means county of residence is less socially vulnerable [13]. Odds ratios (OR) and 95 % confidence intervals (CI) were reported, and factors with an OR > 1.150 or < 0.850 are discussed. Tests for statistical significance were not conducted because these data represent the U.S. population and were not based on population samples. Descriptive analyses were performed for all input variables, and a histogram of total count of initial booster doses administered by month was constructed. This study was reviewed by CDC and conducted consistent with applicable federal law and CDC policy (45C.F.R. part 46.102(1)(2), 21C.F.R. part 56; 42 U.S.C. §241(d); 5 U.S.C. §552a; 44 U.S.C. §3501).

## 3. Results

A total of 168,843,610 persons who completed the primary series of a COVID-19 vaccine by October 31, 2021 were included in the analyses; 95,581,047 (56.6 %) received an initial booster dose and 73,262,563 (43.4 %) did not receive a booster dose by October 31, 2022 (Supplemental Table 1). Model 1 examined factors associated with initial booster dose received ≥ 12 months after primary series completion among those who had completed primary series by October 31, 2021 (Table 1).

Of the 95,581,047 who received their booster dose, 5,079,107 (5.31 %) received the initial booster ≥ 12 months after primary series completion. Increased likelihood of receiving a late first booster dose was associated with receipt of Janssen vaccine, age 12–17 years, Hispanic ethnicity, and American Indian/Alaska Native, Black, or other/unknown race. Compared with those aged 18–34 years, those aged 35–49 years (OR = 0.806, 95 % CI: 0.804–0.809), 50–64 years (OR = 0.805, 95 % CI: 0.803–0.807), and ≥ 65 years (OR = 0.832, 95 % CI: 0.830–0.834) were less likely to have had a late booster.

Model 2 examined factors associated with a missing booster dose among those who had completed the primary series by October 31, 2021 (Table 1). Increased likelihood of not having received a booster dose was associated with receipt of Janssen vaccine as primary series, Hispanic ethnicity, American Indian/Alaska Native, Black, or other/unknown race, and residence in medium or small metro areas, rural areas, high SVI tertile counties, or the South Region. Females, Asian and Pacific Islanders, and persons living in the Pacific or Midwest Regions were more likely to have received a booster dose. Compared with those aged 18–34 years, those aged 35–49 years (OR = 0.684, 95 % CI:0.684–0.685), 50–64 years (OR = 0.430, 95 % CI: 0.430–0.431), and ≥ 65 years (OR = 0.268, 95 % CI: 0.268–0.268) were less likely to have a missing booster dose.

**Fig. 1.**
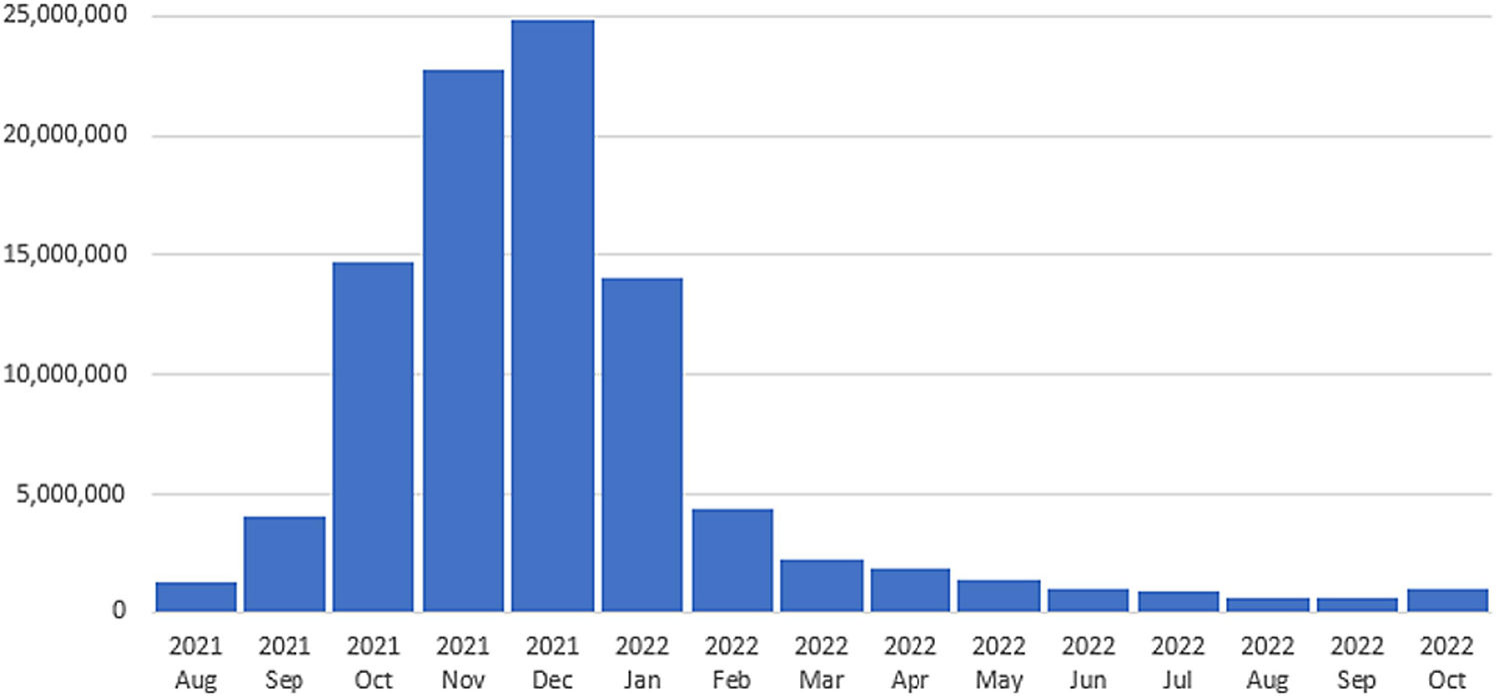
Number of initial COVID-19 booster dose vaccinations among persons ≥ 12 years who had completed primary series by October 31, 2021 by month of administration, August 2021–October 2022, United States.

Fig. 1 presents the reported number of initial booster dose vaccinations among the eligible population by month of administration from August 2021 through October 2022, which peaked in December 2021, then decreased overtime until August 2022, and then increased slightly in October 2022. Supplemental Table 1 presents details on each input variable by timing of interval between completion of primary series and initial booster administration.

## 4. Discussion

The logistic regression models in this study identified several subpopulations who received their initial booster ≥ 12 months after primary series completion or had not received a booster dose. In general, initial booster uptake surged in Autumn 2021 and demand decreased dramatically after January 2022. The peak in booster doses administered overlapped with the Omicron case surge and aligned with initial availability of the first monovalent booster for the public [5,14]. Our results provided information on timing of initial booster vaccination uptake, as a potential reference to inform planning for updated COVID-19 vaccine releases in future [10].

More than 40 % of eligible persons aged ≥ 12 years did not get their initial monovalent booster dose by October 31, 2022. Lower booster uptake was observed among persons living in rural areas, socially vulnerable areas, and in the south U.S. states. Surveys have indicated that the main reasons for not getting boosters have included belief that COVID vaccine boosters were not needed and vaccine safety concerns [15]. In addition, COVID-19 boosters may have been less available in some rural or socially vulnerable areas [16]. Potential strategies to increase COVID-19 vaccination uptake in rural and socially vulnerable communities include increasing vaccine confidence among rural communities through consistent and transparent communication strategies and campaigns, increasing access, and creating sustainable systems to maintain access to vaccines for rural and frontier populations [17].

The choice of COVID-19 vaccine primary series vaccine brand was associated with delayed or missing initial booster dose (monovalent or bivalent). Consistent with previous studies, Janssen primary series recipients were less likely to receive a booster dose [4,18]. Previous reports suggest that the single shot was especially appealing to persons who have less ability to take time away from work or who were in rural settings where access and time commitment may be more prohibitive [19]. In addition, persons with needle fears may have preferred a single dose preparation and been less likely to pursue a booster [20]. Janssen vaccination was also found to be associated with thrombotic thrombocytopenia syndrome (TTS), leading to CDC preferentially recommending mRNA vaccines [21]. Further, Janssen vaccine use as a booster was not recommended and no Janssen bivalent vaccine was manufactured [21].

The results are consistent with findings from surveys suggesting that younger age groups and certain racial and ethnic groups (e.g., Hispanic, non-Hispanic American Indian/Alaska Native, non-Hispanic Black) were more likely to miss or delay COVID-19 vaccination [19,22-24]. Age was found to be strongly associated with delayed or missing initial booster dose, which may have been influenced by age-based COVID-19 vaccine recommendations and by potential elevated risk of COVID-19 related hospitalization and death among older adults with comorbidities [25,26]. Younger age groups and some racial and ethnic groups may have had greater rates of COVID-19 infection prior to booster availability, and their having experienced previous natural infection may have contributed to lower uptake as previous infection was associated with lower uptake in other surveys [22,27,28]. We also found that women were more likely to have a delayed booster but less likely to miss having a booster. Others have found that women may have slightly higher vaccine hesitancy due to pregnancy, fertility, and breastfeeding [29]. However, our results aligned with other booster statistics showing the actual uptake of booster is still higher among women [30,31].

This study had at least four limitations. First, race and ethnicity was not reported or was reported as unknown in more than 20 % of records. Second, the identification and match of a person’s vaccinations, among those who got two or more doses, depended on the link within the jurisdiction-specific immunization information systems. Persons who received a booster dose (monovalent or bivalent) in a different jurisdiction from that of their primary doses could not be represented accurately in these results. Third, there are other factors associated with delayed completion of initial booster dose or missing booster dose that are not available in the national vaccine administration data. Future studies may include other potential social and behavioral factors such as personal attitudes and beliefs. Lastly, our study only focused on persons who completed the 1- or 2-dose primary series; due to data limitations, we were not able to parse out the small percentage of people who were immunocompromised and needed an additional dose for primary series completion, hence their additional dose may have been treated as the initial booster in the present study. Initial boosters during the study period (August 1, 2021 - October 31, 2022) included both monovalent and bivalent boosters; with CDC recommendations focused on the bivalent booster since September 2022, future studies may focus on examining timing and factors associated with uptake of only the bivalent booster. However, this study identified demographic characteristics at the national level of those who did not receive a booster dose or delayed their receipt of a booster dose.

Efforts to improve vaccine confidence overall and in anticipation of future updated COVID-19 vaccines may help to improve uptake, including in populations with lower uptake of COVID-19 vaccine boosters [32]. Our two models had generally similar findings for the demographic factors associated with delayed or missing booster dose. In sum, our data would indicate that persons of younger age, racial and ethnic minority groups, Janssen COVID-19 vaccine recipients, and residents in rural, more socially vulnerable areas, or in the South Region may benefit the most from public health outreach efforts to achieve timely COVID-19 vaccination completion in future.

## Supplementary Material

Supplementary Material

## Figures and Tables

**Table 1:** Factors associated with receipt of an initial booster dose (monovalent or bivalent) ≥ 12 months after the completion of primary series of a COVID-19 vaccine and non-receipt of a booster dose, August 2021- October 2022.

	Model 1: (n = 95,581,047)		Model 2: (n = 168,843,610)	
		
Variable	Had an initial COVID- 19 monovalent booster ≥ 12-month after the completion of primary series		Did not receive a COVID-19 monovalent or bivalent booster	
		
	*OR*	*95 %CI*	*OR*	*95 %CI*
**Vaccine Type (Series Completion)**				
**Record**†)				
Janssen	1.173*	1.169–1.178	1.679*	1.677–1.681
Moderna	0.973	0.971–0.975	0.909	0.908–0.910
Pfizer-BioNTech	Ref		Ref	
**Age Group (in years)**				
12–17	1.406*	1.400–1.411	1.061	1.059–1.062
18–34	Ref		Ref	
35–49	0.806*	0.804–0.809	0.684*	0.684–0.685
50–64	0.805*	0.803–0.807	0.430*	0.430–0.431
65+	0.832*	0.830–0.834	0.268*	0.268–0.268
**Sex**				
Female	1.062	1.061–1.064	0.844*	0.844–0.845
Male	Ref		Ref	
**Race and Ethnicity**				
Hispanic/Latino	1.212*	1.208–1.215	1.585*	1.583–1.586
Non-Hispanic Black	1.298*	1.293–1.302	1.239*	1.237–1.241
Non-Hispanic AI/AN	1.560*	1.545–1.575	1.258*	1.253–1.263
Non-Hispanic Asian/OPI	0.947	0.943–0.951	0.736*	0.735–0.737
Other/Unknown	1.187*	1.184–1.189	1.209*	1.208–1.210
Non-Hispanic White	Ref		Ref	
**Urbanicity**				
Non-metro	1.037	1.034–1.040	1.359*	1.357–1.360
Medium/Small metro	1.043	1.041–1.045	1.163*	1.162–1.164
Large metro	Ref		Ref	
**Social Vulnerability Index** §				
High	1.074	1.071–1.076	1.228*	1.225–1.231
Medium	1.043	1.041–1.045	1.103	1.100–1.105
Low	Ref		Ref	
**Region** **				
Pacific	0.936	0.933–0.938	0.666*	0.665–0.667
Midwest	0.891	0.889–0.894	0.827*	0.826–0.828
South	1.090	1.088–1.093	1.279*	1.278–1.280
Mountain	0.966	0.962–0.970	0.914	0.912–0.915
Northeast	Ref		Ref	

* Factors with an Odds Ratio > 1.150 or < 0.850.

† The vaccination brand of the series completion dose (e.g., the brand of the second dose of a mRNA primary vaccine).

§ More details about Social Vulnerability Index: https://www.atsdr.cdc.gov/placeandhealth/svi/index.html.

** For this study, Region classifications were chosen to align with the classifications used in other publications of this group: South Region includes the U.S. Census South Atlantic and South Central Regions, and the southern states of the U.S. Census Mountain Region: AZ, NM, OK, AR, LA, MS, AL, TN, KY, GA, SC, NC, WV, MD, VA, FL, DE, & DC; Midwest Region includes the U.S. Census North Central Regions: ND, SD, NE, KS, MN, IA, MO, IL, WI, IN, MI, & OH; Mountain Region includes the northern states of the U.S. Census Mountain Region: NV, UT, CO, WY, MT, ID; Pacific Region includes WA, HI, AK, OR, & CA; Northeast Region includes PA, NY, VT, NH, ME, MA, RI, CT, & NJ.

**Abbreviations;** CI = Confidence Interval; AI/AN=American Indian/Alaska Native; OPI = Other Pacific Islander

## Data Availability

The authors do not have permission to share data.

## Footnotes

a IISs are confidential, computerized, population-based systems that collect and consolidate vaccination data from providers in 64 public health jurisdictions and can be used to track administered vaccines and measure vaccination coverage. The 64 IIS jurisdictions comprise the 50 U.S. states, eight U.S. territories and freely associated states (Puerto Rico, U.S. Virgin Islands, American Samoa, Northern Mariana Islands, Guam, Marshall Islands, Palau, and the Federated States of Micronesia), and six local jurisdictions (Chicago, Illinois; District of Columbia; Houston, Texas; New York, New York; Philadelphia, Pennsylvania; and San Antonio, Texas). Texas data for persons in all age groups and Idaho data for persons aged 12–17 years were not available due to data-sharing restrictions.

b For this study, Region classifications were chosen to align with the classifications used in other publications of this group: South Region includes the U. S. Census South Atlantic and South Central Regions, and the southern states of the U.S. Census Mountain Region: AZ, NM, OK, AR, LA, MS, AL, TN, KY, GA, SC, NC, WV, MD, VA, FL, DE, & DC; Midwest Region includes the U.S. Census North Central Regions: ND, SD, NE, KS, MN, IA, MO, IL, WI, IN, MI, & OH; Mountain Region includes the northern states of the U.S. Census Mountain Region: NV, UT, CO, WY, MT, ID; Pacific Region includes WA, HI, AK, OR, & CA; Northeast Region includes PA, NY, VT, NH, ME, MA, RI, CT, & NJ.

c Large fringe metro counties are counties in Metropolitan Statistical Areas of 1 million or more population that do not qualify as large central. For more information regarding urbanicity classification, please see citation 7.
