# Supplementary material for: Social and demographic factors associated with receipt of a COVID-19 vaccine initial booster dose and with interval between primary series completion and initial booster dose uptake among persons aged ≥ 12 years, United States, August 2021-October 2022
Source: Vaccine. Author manuscript; Available in PMC 2024 Jun 20. (PMC11187615; doi:10.1016/j.vaccine.2024.02.089)
Supplement: Supplementary Material [file NIHMS1999688-supplement-Supplementary_Material.docx]

**Supplemental Table**. COVID-19 vaccine initial booster (monovalent and bivalent) dose timing and dose completion by sociodemographic factors among persons aged ≥12 years who had completed a primary series, United States, August 2021-October 2022

| **Variable** | **Initial Booster dose timing** | | | | | | | | | | **Total (N)** |
| --- | --- | --- | --- | --- | --- | --- | --- | --- | --- | --- | --- |
|  | ***Less than 6 months*** | ***%*** | ***6 to 9 months*** | ***%*** | ***9-12 months*** | ***%*** | ***More than 12 months*** | ***%*** | ***Missing booster*** | **%** |  |
|  |  |  |  |  |  |  |  |  |  |  |  |
| **Total** | 4,077,407 | 2.41 | 69,707,447 | 41.29 | 16,717,086 | 9.9 | 5,079,107 | 3.01 | 73,262,563 | 43.39 | 168,843,610 |
| **Vaccine type** |  |  |  |  |  |  |  |  |  |  |  |
| **(Series completion record*)** | |  |  |  |  |  |  |  |  |  |  |
| Janssen | 389,193 | 2.98 | 3,715,908 | 28.43 | 1,147,231 | 8.78 | 338,646 | 2.59 | 7,478,427 | 57.22 | 13,069,405 |
| Pfizer-BioNTech | 2,405,736 | 2.56 | 38,955,088 | 41.45 | 7,988,127 | 8.5 | 2,834,180 | 3.02 | 41,806,529 | 44.48 | 93,989,660 |
| Moderna | 1,282,478 | 2.08 | 27,036,451 | 43.76 | 7,581,728 | 12.3 | 1,906,281 | 3.09 | 23,977,607 | 38.81 | 61,784,545 |
| **Age group (in years)** |  |  |  |  |  |  |  |  |  |  |  |
| 12–17 | 282,167 | 2.55 | 3,304,915 | 29.85 | 574,578 | 5.19 | 373,049 | 3.37 | 6,537,208 | 59.04 | 11,071,917 |
| 18–34 | 712,279 | 1.89 | 10,936,982 | 28.96 | 3,326,216 | 8.81 | 962,477 | 2.55 | 21,826,833 | 57.80 | 37,764,787 |
| 35–49 | 769,233 | 2.13 | 13,094,070 | 36.26 | 3,706,979 | 10.3 | 914,547 | 2.53 | 17,626,336 | 48.81 | 36,111,165 |
| 50–64 | 1,196,982 | 2.84 | 19,231,929 | 45.70 | 4,407,357 | 10.5 | 1,295,442 | 3.08 | 15,954,382 | 37.91 | 42,086,092 |
| 65+ | 1,116,746 | 2.67 | 23,139,551 | 55.35 | 4,701,956 | 11.2 | 1,533,592 | 3.67 | 11,317,804 | 27.07 | 41,809,649 |
| **Sex** |  |  |  |  |  |  |  |  |  |  |  |
| Female | 1,895,377 | 2.12 | 37,620,833 | 42.09 | 9,861,930 | 11 | 2,862,226 | 3.2 | 37,151,423 | 41.56 | 89,391,789 |
| Male | 2,182,030 | 2.75 | 32,086,614 | 40.38 | 6,855,156 | 8.63 | 2,216,881 | 2.79 | 36,111,140 | 45.45 | 79,451,821 |
| **Race and ethnicity** |  |  |  |  |  |  |  |  |  |  |  |
| Hispanic/Latino | 523,728 | 2.49 | 6,403,966 | 30.43 | 1,865,965 | 8.87 | 574,416 | 2.73 | 11,674,812 | 55.48 | 21,042,887 |
| Non-Hispanic Black | 427,936 | 3.30 | 4,540,301 | 35.06 | 1,195,395 | 9.23 | 428,335 | 3.31 | 6,358,423 | 49.10 | 12,950,390 |
| Non-Hispanic AI/AN | 30,418 | 2.58 | 332,627 | 28.18 | 151,414 | 12.8 | 44,875 | 3.8 | 621,186 | 52.62 | 1,180,520 |
| Non-Hispanic Asian/OPI | 213,641 | 2.22 | 4,839,681 | 50.19 | 1,021,520 | 10.6 | 299,938 | 3.11 | 3,267,699 | 33.89 | 9,642,479 |
| Non-Hispanic White | 1,929,534 | 2.40 | 36,570,117 | 45.53 | 7,688,754 | 9.57 | 2,351,801 | 2.93 | 31,780,582 | 39.57 | 80,320,788 |
| Other/unknown | 952,150 | 2.18 | 17,020,755 | 38.94 | 4,794,038 | 11 | 1,379,742 | 3.16 | 19,559,861 | 44.75 | 43,706,546 |
| **Urbanicity** |  |  |  |  |  |  |  |  |  |  |  |
| Non-metro | 454,627 | 2.30 | 7,583,429 | 38.38 | 2,066,079 | 10.5 | 558,338 | 2.83 | 9,096,283 | 46.04 | 19,758,756 |
| Medium/small metro | 1,183,897 | 2.39 | 19,850,219 | 40.08 | 4,852,536 | 9.8 | 1,482,262 | 2.99 | 22,156,869 | 44.74 | 49,525,783 |
| Large metro | 2,438,883 | 2.45 | 42,273,799 | 42.46 | 9,798,471 | 9.84 | 3,038,507 | 3.05 | 42,009,411 | 42.20 | 99,559,071 |
| **Social Vulnerability Index**^†^ |  |  |  |  |  |  |  |  |  |  |  |
| High | 1,271,790 | 2.53 | 18,672,687 | 37.09 | 5,218,577 | 10.4 | 1,506,507 | 2.99 | 23,673,591 | 47.02 | 50,343,152 |
| Medium | 1,642,675 | 2.42 | 28,030,394 | 41.29 | 6,697,938 | 9.87 | 2,057,739 | 3.03 | 29,456,336 | 43.39 | 67,885,082 |
| Low | 1,162,942 | 2.30 | 23,004,366 | 45.45 | 4,800,571 | 9.48 | 1,514,861 | 2.99 | 20,132,636 | 39.78 | 50,615,376 |
| **Region**^‡^ |  |  |  |  |  |  |  |  |  |  |  |
| Pacific | 889,145 | 2.69 | 14,978,160 | 45.25 | 3,896,783 | 11.8 | 1,079,833 | 3.26 | 12,257,868 | 37.03 | 33,101,789 |
| Midwest | 798,157 | 2.18 | 16,750,144 | 45.76 | 3,788,534 | 10.4 | 1,057,167 | 2.89 | 14,208,417 | 38.82 | 36,602,419 |
| South | 1,371,063 | 2.47 | 19,809,644 | 35.69 | 4,855,616 | 8.75 | 1,636,293 | 2.95 | 27,831,794 | 50.14 | 55,504,410 |
| Mountain | 195,399 | 2.31 | 3,528,795 | 41.78 | 814,879 | 9.65 | 248,317 | 2.94 | 3,657,945 | 43.31 | 8,445,335 |
| Northeast | 823,643 | 2.34 | 14,640,704 | 41.61 | 3,361,274 | 9.55 | 1,057,497 | 3.01 | 15,306,539 | 43.50 | 35,189,657 |

**Footnote:**

* Vaccination brand of series completion dose (e.g., brand of second dose of an mRNA primary vaccine).

† More details about Social Vulnerability Index: <https://www.atsdr.cdc.gov/placeandhealth/svi/index.html>

‡ Region classifications were chosen to align with classifications used in other publications of this group: South Region includes U.S. Census South Atlantic and South Central Regions, and southernmost states of the U.S. Census Mountain Region: AZ, NM, OK, AR, LA, MS, AL, TN, KY, GA, SC, NC, WV, MD, VA, FL, DE, & DC; Midwest Region includes U.S. Census North Central Regions: ND, SD, NE, KS, MN, IA, MO, IL, WI, IN, MI, & OH; Mountain Region includes northern states of the U.S. Census Mountain Region: NV, UT, CO, WY, MT, ID; Pacific Region includes WA, HI, AK, OR, & CA; Northeast Region includes PA, NY, VT, NH, ME, MA, RI, CT, & NJ.

**Abbreviations:** AI/AN=American Indian/Alaska Native; OPI = Other Pacific Islander
